# Determining the psychometric properties of the Enhancing Decision-making Assessment in Midwifery (EDAM) measure in a cross cultural context

**DOI:** 10.1186/s12884-016-0882-3

**Published:** 2016-04-28

**Authors:** Elaine Jefford, Julie Jomeen, Colin R. Martin

**Affiliations:** Southern Cross University, School of Health and Human Sciences, Coffs Harbour, Australia; University of Hull, Faculty of Health and Social Care, Hull, UK; Buckinghamshire New University, 106 Oxford Road, Uxbridge, UB8 1NA UK

**Keywords:** Midwifery, Decision-making, Assessment, Psychometric, Cross-cultural, birth, clinical Reasoning, midwifery practice

## Abstract

**Background:**

The ability to act on and justify clinical decisions as autonomous accountable midwifery practitioners, is encompassed within many international regulatory frameworks, yet decision-making within midwifery is poorly defined. Decision-making theories from medicine and nursing may have something to offer, but fail to take into consideration midwifery context and philosophy and the decisional autonomy of women. Using an underpinning qualitative methodology, a decision-making framework was developed, which identified *Good Clinical Reasoning* and *Good Midwifery Practice* as two conditions necessary to facilitate optimal midwifery decision-making during 2nd stage labour. This study aims to confirm the robustness of the framework and describe the development of Enhancing Decision-making Assessment in Midwifery (EDAM) as a measurement tool through testing of its factor structure, validity and reliability.

**Method:**

A cross-sectional design for instrument development and a 2 (country; Australia/UK) x 2 (Decision-making; optimal/sub-optimal) between-subjects design for instrument evaluation using exploratory and confirmatory factor analysis, internal consistency and known-groups validity. Two ‘expert’ maternity panels, based in Australia and the UK, comprising of 42 participants assessed 16 midwifery real care episode vignettes using the empirically derived 26 item framework. Each item was answered on a 5 point likert scale based on the level of agreement to which the participant felt each item was present in each of the vignettes. Participants were then asked to rate the overall decision-making (optimal/sub-optimal).

**Findings:**

Post factor analysis the framework was reduced to a 19 item EDAM measure, and confirmed as two distinct scales of ‘*Clinical Reasoning’* (CR) and ‘*Midwifery Practice*’ (MP). The CR scale comprised of two subscales; ‘the clinical reasoning process’ and ‘integration and intervention’. The MP scale also comprised two subscales; women’s relationship with the midwife’ and ‘general midwifery practice’.

**Conclusion:**

EDAM would generally appear to be a robust, valid and reliable psychometric instrument for measuring midwifery decision-making, which performs consistently across differing international contexts. The ‘women’s relationship with midwife’ subscale marginally failed to meet the threshold for determining good instrument reliability, which may be due to its brevity. Further research using larger samples and in a wider international context to confirm the veracity of the instrument’s measurement properties and its wider global utility, would be advantageous.

**Electronic supplementary material:**

The online version of this article (doi:10.1186/s12884-016-0882-3) contains supplementary material, which is available to authorized users.

## Background

A midwife has a scope of practice that is built on the International Confederation of Midwives (ICM) international definition of the midwife [1], which recognises the midwife as a responsible and accountable professional who works in partnership with women to give the necessary support, care and advice during pregnancy, labour and the postpartum period. Midwifery scope of practice is defined by essential competencies, which are the combination of knowledge, psychomotor, communication and decision-making skills that enable an individual to perform a specific task to a defined level of proficiency.

Midwives’ ability to act on and justify clinical decisions as an autonomous accountable practitioner, is encompassed within the regulatory frameworks of countries across the globe. The Nursing and Midwifery Board of Australia and the United Kingdom (UK) Nursing and Midwifery Council [2–4], provide examples of competency standards related to decision-making. These competency standards refer to midwives providing advice to facilitate decision-making by the woman, and/or involving the woman in decision-making. Yet in these documents ‘decision-making’, is not explicitly defined. Women’s participation in decision-making is a growing expectation in maternity care [5]. Maternity care providers use their expertise to help women interpret information and share the best available evidence to support the woman to consider options and achieve informed preferences [6, 7]. In a maternity context, particularly during labour and birth, where the need to make decisions can be time limited and where women may be distracted by pain and contractions, the level of interaction necessary to promote decisional autonomy for the woman, or shared decision-making may be compromised [5].

Whilst nursing and medicine have discipline specific decision-making processes [8–11], their usefulness to midwifery has been questioned. Though some of the detailed decision-making theories have something to offer midwifery they fail to take into consideration the context and philosophy within which midwifery is practised and the decisional autonomy afforded to women [12–15]. Midwives have to consider both the woman and the baby; not as separate entities but as an indivisible whole. Hence, midwifery decision-making should incorporate both objective and subjective elements, including the context of decision-making and the emotions and intuitions of both the woman and the midwife.

Globally, and for differing reasons, research shows there is great variation in midwife decision-making at the time of birth and that failure to actively engage in effective clinical decision-making is an identified culprit in substandard care, particularly in labour, which affects the safety and quality of midwifery care for women and babies at a global level [13–17].

### Frameworks for supporting and evaluating midwifery decision-making

Midwifery decision-making frameworks are offered by the ICM ‘Clinical Decision-Making Framework’ and by some regulatory bodies, including the ‘Practice Decisions Flowchart and Midwifery Practice’ [18]. Page and Hutton [19] offer five steps of evidence-based midwifery based upon the midwife/women partnership, promoting a continuity of care model of midwifery. In such models the midwife has time to know the woman and/or there is time to talk decisions through. Unfortunately this is not a context within which all midwives practise or women receive care. In addition, it has been proposed that these frameworks/principles lack details on the clinical reasoning process, as used within medicine and nursing, which midwives also need to undertake clinical decision-making [13]. Indeed, a general paucity of evidence considers midwifery decision-making in either education or clinical practice and studies demonstrate an absence of any steps of clinical reasoning as part of that process [14]. The potential negative consequences of this are that midwifery decision-making is less easy to consensually validate with fellow clinicians [14] but also difficult to evaluate. Further, teaching and learning on decision-making is much less effective due to lack of clear and specific steps [20–23]. Ultimately the lack of a clear and consistent framework may undermine midwives’ ability to adhere to the midwifery regulatory ‘Framework’ [2–4] and associated legal requirements.

In response to the lack of a clear framework for decision-making incorporating clinical reasoning and specifically orientated to a midwifery context, a framework was developed using bidirectional analysis of theory and data drawn from midwives within the Australian clinical environment and has been described fully elsewhere [14, 15, 24]. The underpinning study interviewed 26 Australian midwives with a focus on eliciting narratives about decision-making in second stage labour. No previous study has systematically explored the processes midwives undertake in order to reach a clinical decision in second stage labour. Interviewees were invited to tell two narratives as examples of their decision-making and subsequent actions; one they thought of as a positive example and the other as a negative example of decision-making. The narratives were analysed and interpreted in the light of extant literature. This approach used induction (from the data) and deduction (from the literature) and facilitated synthesis of both experiential and theoretical knowledge of clinical decision-making, as the foundations of a framework.

**Fig. 1:**
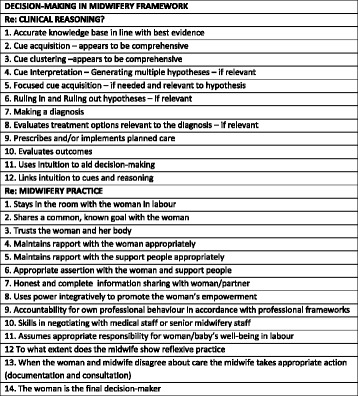
Decision-making framework constructs and associated factors

This framework identifies two necessary and sufficient conditions required to facilitate optimal midwifery decision-making during 2nd stage labour. Factors, which may or may not influence the final decision, were placed under two identified conditions of ***Good Clinical Reasoning*** [24] and ***Good Midwifery Practice.*** Variables, considerations, actions and behaviours necessary to fulfil those two essential conditions, emerging from the interviews, were incorporated into a framework (Fig. 1). As with any framework, its utility in practice is essential. This framework, if determined to be a robust measurement tool could be used to determine the effectiveness of educational and/or clinical training packages on clinical decision-making or to guide self-reflection on decision-making. There would be ultimate value in a tool that could guide decision-making in practice and evaluate it in relation to clinical outcomes.

Whilst a decision-making framework would provide valuable criteria against which to judge the validity and professional appropriateness of midwifery decision-making and ability to relate such data to clinical outcomes, this requires confirmation of the robustness of the framework and it’s utility as a potential measurement tool. The aim of the present study was to determine the utility of the qualitatively derived framework as an assessment tool and consider its factor structure, validity and reliability.

## Methods

### Design

The study adopted a sequential instrument development mixed-method design [25]. The qualitative aspect has been reported elsewhere [15, 24]. Presented here is the quantitative component of the study which represented two study designs nested within a common data set utilised for both instrument development and evaluation. These were a cross-sectional design for instrument development and a 2 (country; Australia/UK) x 2 (Decision-making; optimal/sub-optimal) between-subjects design for instrument evaluation. The study was approved by the Ethics Committees of the University of Canberra and the University of Hull.

### Participants

Participants were convened into two ‘expert’ maternity panels, based in Australia and the UK, chosen for the shared similarities in professional frameworks and midwifery philosophy, yet apparent cultural differences in models of care delivery. In the UK care is almost exclusively delivered within the NHS. Women in the UK are offered both choice of place of birth and choice of lead carer. Care from a midwife, as the lead professional throughout pregnancy, birth and the postnatal period is however the default model of care for low risk women, with obstetric care usually only provided for women considered high risk. Though both midwives and obstetricians do offer private care, in the UK, this applies to a relatively small number of women. In Australia, there is nationally funded, universal health care. This is known as public care and in the maternity context this is provided in a variety of models depending on the location and type of health care service. Predominantly, maternity care is managed by doctors/obstetricians (regardless of the level of risk of the women) with midwives supporting that care. Increasingly, publically funded health services are offering midwife-led continuity of care models. Private practice midwives in Australia also offer women the chance to experience one on one midwifery care. If women choose to, they can pay through private health insurance or their own resources to access a private obstetrician, who will manage their care throughout. Policy in Australia now promotes collaboration between midwives and doctors as an important component of achieving positive maternity outcome.

The panels were recruited through a process of advertising via professional organisations and midwifery discussion forums. Interested parties were asked to submit an expression of interest and outline relevant expertise to determine eligibility for the study. Selection of the panels was based on representation from across the different models of service provision within the two countries, significant experience in a clinical context, as well as consumer representation. Each panel also included academic midwives. The UK panel specifically sought to include supervisors of midwives and consultant midwives, to reflect the UK context. Unfortunately the UK independent midwife and consumer representative initially recruited withdrew from the study due to time pressures. The Australian panel comprised of 25 members and the UK group consisted of 17 members and there was good representation of midwives working in differing clinical contexts with varied but significant levels of experience (see Additional file 1).

### Procedure

Following expression of interest, potential participants were provided with an information leaflet and a consent form via email. Following consent, participants were sent a study pack. Each pack consisted of 16 midwifery real care episode vignettes, derived from the interviews with midwives undertaken in the qualitative study [14, 15, 24] and 16 framework questionnaires. The order of presentation of the vignettes was consistent within the packs. Panel members were asked to complete a questionnaire based on the framework in for each vignette (see Additional file 2 [14]). Participants responded to each of the questionnaire items on a 5 point likert scale based on the level to which they agreed or disagreed that each element was present in each of the vignettes. In addition they were asked at the end to determine whether they perceived the scenario the vignette represented:

- Good decision-making and Good midwifery practice (optimal decision-making)
- Good decision-making and Poor midwifery practice (sub-optimal decision-making)
- Poor decision-making and Good midwifery practice (sub-optimal decision-making)
- Poor decision-making and Poor midwifery practice (sub-optimal decision-making)

In the underpinning qualitative study 52 narratives of midwives decision-making were captured. The 16 vignettes for this study were chosen to ensure representation of each of the above decision-making categories. Four examples of each were utilised. The aim was that each participant would complete 16 questionnaires, leading to a total anticipated response rate of 672 questionnaires. Participants were also asked to make comment on the clarity and relevance of the individual items within the framework as they worked through the questions in relation to each vignette.

### Statistical analysis

The development and optimisation of the clinical reasoning scale (CR) and the midwifery practice (MP) scale was based on the classical approach to instrument development described by Kline [26]. This two-stage approach utilised an initial principal components analysis (PCA) with an oblimin rotation [26] to determine the underlying structure of each scale and select the optimal number of items within each scale and associated sub-scales. Oblimin rotation was selected in view of the possibility of components within each measure being correlated [26]. A substantive item-component loading was based on the criterion of a greater than 0.4 loading and less than 0.3 on any other extracted component [26]. Following initial structure determination a confirmatory factor analysis (CFA; [26, 27]) was conducted on a second data set using a random-split approach to corroborate the findings from the initial structural determination and to establish model fit characteristics (Byrne, 2010).

Consistent with the CFA approach of others [28–30], a maximum-likelihoods (ML) estimation was adopted. Multiple goodness of fit tests [31], specifically, the comparative fit index (CFI; [31, 32]) and the root mean squared error of approximation (RMSEA; [28]) were used to evaluate model fit. A CFI greater than 0.90 indicates an acceptable fit to the data [33] and a more stringent CFI value equal to or greater than 0.95 is indicative of good model fit [34]. RMSEA values of less than or equal to 0.08 indicate acceptable data fit [35] and values of less than or equal to 0.05 indicate a good fit to the data [36]. A statistically significant χ^2^ indicates a significant proportion of variance within the data is unexplained by the model [31], however, a significant χ^2^ statistic is not uncommon as an effect of trivial variations in data [33], thus model evaluation is best based on fit statistics such as CFI and RMSEA [28, 37].

A further objective of the study concerns determining the measurement and structural invariance [28] of the developed measures. Invariance refers to the desirable characteristic of an instrument performing in a consistent manner across different groups, thus assuring the tool is measuring fundamentally the same construct, in the same way [28, 38]. Applying this directly to the CR and MP scales, a key focus would be to determine that the measures are invariant between countries (Australia and UK). This supports the conclusion that any differences in scores found as a function of country are real differences, rather than an artefact of the measure being confounded by one group responding to the measure in a characteristically different way due to instrument measurement anomaly. Evaluating the measurement and structural invariance of the tool thus serves an important role in appraising instrument validity and transferability.

Statistical analysis were conducted using PASW version 18 (SPSS, [39, 40]) and Analysis of Moment Structures (AMOS) version 18 [41].

Known-groups validity was evaluated by testing for differences in CR and MP scales and potential sub-scale scores as a function of participant vignette judgement based on a binary decision-making outcome (optimal/sub-optimal). Given the differences in both culture and service format between Australia and the UK, as previously described, which might impact on a midwives’ ability to function as the key decision-maker or work in partnership with women, the sensitivity of the CR and MP scales was also evaluated in this analysis. It was predicted that CR and MP scale scores would be significantly higher in those participants making an optimal decision-making vignette judgement compared to those making a sub-optimal decision-making judgement.

Internal consistency of the CR and MP scales was conducted to determine the measure reached criteria for clinical and research purposes using the Cronbach coefficient alpha statistical procedure. Cronbach’s alpha of 0.70 is accepted as a minimum for internal reliability [26, 27].

## Results

### Data management

Six-hundred and seventy-two ‘cases’ were computed from participant data of which 400 cases were Australian (68 %). Fifty-eight (8.6 %) had completely missing data and these cases were removed from the data set. A further 144 cases (21.4 %) had partially missing data from either the CR or MP scale. Where a case had more than two items missing from all items (thus greater than 10 %), these were removed from the remaining data set (*N* = 25, 4.1 %). The remaining incomplete (<10 % missing) cases of the data set (*N* = 119, 20.2 %) were then subjected to the expectation-maximisation (EM) approach to substitute values for the missing items. The EM approach represents a robust method of dealing with missing data where there are few missing items from both a case and data set. The data set was then subjected to a random split procedure to furnish two data sets, one for PCA (*N* = 304) and one for CFA (285).

### Principal components analysis

The mean and standard deviation of the CR and MP scale items are shown in Table 1. The CR items were subjected to PCA first. The Kaiser-Meyer-Olkin (KMO) measure of sampling adequacy and the Bartlett Test of Sphericity (BTS) were conducted to ensure that the characteristics of the clinical reasoning dataset were suitable for the PCA to be conducted [42, 43]. KMO analysis yielded an index of 0.92, and in combination with a highly significant BTS, χ^2^_(df = 66)_ = 2969.82, *p* < 0.001, confirmed that the data distribution satisfied the psychometric criteria [42, 43]. Two components with eigenvalues greater than 1 emerged, the two component solution cumulatively accounted for 70 % of the total variance. The item-component loadings of the individual CR items are shown in Table 2. Clear differentiation of components by item loadings was observed and just one cross-loading item (item-7) rejected.

**Table 1:** Mean and standard deviation (SD) of clinical reasoning and midwifery practice items

tem	Mean	SD
Clinical Reasoning		
CR1	2.73	1.09
CR2	2.83	1.04
CR3	2.77	1.05
CR4	2.55	1.09
CR5	2.59	1.07
CR6	2.52	1.07
CR7	2.79	1.04
CR8	2.53	1.18
CR9	2.75	1.07
CR10	2.67	1.16
CR11	2.82	1.10
CR12	2.64	1.18
Midwifery Practice		
MP1	3.18	1.11
MP2	2.92	1.01
MP3	3.19	0.91
MP4	2.82	1.15
MP5	2.50	1.03
MP6	2.41	1.26
MP7	2.15	1.30
MP8	2.36	1.28
MP9	2.56	1.30
MP10	1.88	1.05
MP11	2.54	1.29
MP12	2.74	1.20
MP13	1.87	0.69
MP14	2.34	1.34

**Table 2:** Item-component loadings of clinical reasoning items following PCA and oblimin rotation

Item number	Component one	Component two
1	**0.76**	−0.10
2	**0.87**	−0.02
3	**0.86**	0.07
4	**0.84**	0.09
5	**0.81**	0.11
6	**0.71**	0.17
7	0.35	0.53
8	0.27	**0.64**
9	0.09	**0.68**
10	0.14	**0.69**
11	−0.18	**0.98**
12	0.04	**0.84**

Clearly differentiated and substantive (>0.4) item-component loadings are shown in bold

Examination of individual item content suggest the first CR component represents a subscale of ‘clinical decision-making process’ and the second CR component seemingly representing a subscale of ‘intervention and integration’.

For the MP scale, the KMO analysis yielded an index of 0.92, and a highly significant BTS, χ^2^_(df = 91)_ = 2336.01, *p* < 0.001, confirming data suitability for PCA. Two components with eigenvalues greater than 1 emerged which accumulatively accounted for 59 % of the total variance. The item-component loadings of the individual MP items are shown in Table 3. Clear differentiation of components by item loadings was generally observed, though items 4, 5, 6 and 8 were cross-loading and thus rejected.

**Table 3:** Item-component loadings of midwifery practice items following PCA and oblimin rotation

Item number	Component one	Component two
1	−0.17	**0.80**
2	0.20	**0.70**
3	0.04	**0.80**
4	0.37	0.64
5	0.47	0.43
6	0.69	0.31
7	**0.71**	0.21
8	0.61	0.36
9	**0.83**	−0.07
10	**0.56**	0.09
11	**0.88**	−0.08
12	**0.79**	−0.14
13	**0.65**	−0.11
14	**0.42**	0.12

Clearly differentiated and substantive (>0.4) item-component loadings are shown in bold

Individual item content suggest the first midwifery practice component represents a subscale of the ‘woman’s relationship with midwife’ and the second midwifery component a subscale of ‘general midwifery practice’.

### Confirmatory factor analysis

The two-factor EFA-derived clinical reasoning scale was evaluated using CFA on the second data set (*N* = 285). Initial model fit was found to be poor based on established acceptability criteria, χ^2^_(df = 43)_ = 451.33, *p* < 0.001, χ^2^/df = 10.50, CFI = 0.86 and RMSEA = 0.18. Examination of model fit indices and removal of comparatively lower factor loading items (items 1 and 11) produced a much improved model with fewer items and good model fit characteristics, χ^2^_(df = 25)_ = 69.99, *p* < 0.001, χ^2^/df = 2.80, CFI = 0.98 and RMSEA = 0.08, with the residual error variance being specified as correlated between items 2 and 3. Similarly, the two-factor EFA-derived midwifery practice scale was evaluated using CFA. Initial model fit was found to be good, χ^2^_(df = 34)_ = 70.03, *p* < 0.001, χ^2^/df = 2.06, CFI = 0.96 and RMSEA = 0.06. Examination of model fit indices suggested no evidence of potential to improve model fit further.

### Evaluation of measurement and structural invariance

The best-fit CR and MP models above were evaluated for measurement and structural invariance to determine the construct robustness and stability of each scale across groups (Australia/UK). Taking the CR scale first, a configural model was developed from which increasingly constrained models were directly evaluated. The first comparison model was the metric model with item-factor loadings specified as being equal between groups, followed by a measurement model, with item-factor loadings and factor variances and covariances being specified equal between groups, and finally, the most constrained model, with item-factor loadings and factor variances and covariances being specified equal between groups, and the covariance between item 2 and 3 residuals being specified as equal. The performance of the increasing constrained models was evaluated against the configural model using the χ^2^ difference (χ^2^diff) test. Evaluation of the CR scale revealed the tool to have excellent measurement invariance characteristics. Similarly, the MP scale was evaluated using the same procedure with the exception that the configural model had no covariances specified between any of the error residuals thus two constrained models only were required for evaluating measurement invariance. The models evaluated are shown in Table 4.

**Table 4:** Comparison of models evaluating measurement and structural invariance of *clinical reasoning* and *midwifery* practice scales across country categorisation (Australia/UK)

Model and scale	χ^2^_(df)_	χ^2^ diff _(df)_	*p*
Clinical reasoning			
Configural	112.43 (50)		
Measurement	116.59 (57)	4.15 (7)	0.76
Structural	119.31 (60)	6.88 (10)	0.74
Most constrained^a^	122.01 (61)	9.57 (11)	0.57
Midwifery Practice			
Configural	113.77 (68)		
Measurement	128.67 (76)	14.89 (8)	0.06
Structural	129.66 (79)	15.89 (11)	0.14

^a^Represents the most constrained model to incorporate covariance of error residuals between item 2 and 3. Note: A non-significant χ^2^ diff is indicative of invariance

### Known-groups validity

The mean scores and standard deviation for the CR and MP scales and associated subscales as a function of decision-making categorisation and country type are shown in Table 5. 2 × 2 ANOVA of the clinical reasoning total score revealed a highly statistically significant main effect of outcome category, *F*_(1, 281)_ = 194.12, *p* < 0.001, no significant main effect of country type, *F*_(1, 281)_ = 2.65, *p* = 0.10, and no significant interaction between outcome category and country type, *F*_(1, 281)_ = 0.95, *p* = 0.87. Similarly, the ‘clinical reasoning process’ subscale revealed a highly statistically significant main effect of outcome category, *F*_(1, 281)_ = 123.32, *p* < 0.001, no significant main effect of country type, *F*_(1, 281)_ = 0.61, *p* = 0.43, and no significant interaction between outcome category and country type, *F*_(1, 281)_ = 0.88, *p* = 0.88. In contrast, the ‘intervention and integration’ subscale revealed a highly statistically significant main effect of outcome category, *F*_(1, 281)_ = 198.84, *p* < 0.001, a significant main effect of country type, *F*_(1, 281)_ = 5.59, *p* = 0.02, and no significant interaction between outcome category and country type, *F*_(1, 281)_ = 0.94, *p* = 0.33. Examination of the MP scale total score revealed a highly statistically significant main effect of outcome category, *F*_(1, 281)_ = 241.06, *p* < 0.001, no significant main effect of country type, *F*_(1, 281)_ = 3.21, *p* = 0.07, and no significant interaction between outcome category and country type, *F*_(1, 281)_ = 0.34, *p* = 0.56. Likewise, the ‘women’s relationship with midwife’ subscale revealed a highly statistically significant main effect of outcome category, *F*_(1, 281)_ = 76.09, *p* < 0.001, no significant main effect of country type, *F*_(1, 281)_ = 0.06, *p* = 0.80, and no significant interaction between outcome category and country type, *F*_(1, 281)_ = 0.01, *p* = 0.99. Finally, the ‘general midwifery practice’ subscale revealed a highly statistically significant main effect of outcome category, *F*_(1, 281)_ = 240.91, *p* < 0.001, a significant main effect of country type, *F*_(1, 281)_ = 4.76, *p* = 0.03, and no significant interaction between outcome category and country type, *F*_(1, 281)_ = 0.56, *p* = 0.45.

**Table 5:** Mean scores and standard deviations for the clinical reasoning and midwifery practice and associated subscales as a function of decision-making categorisation and country type

Scale	Australia	UK
	Sub-optimal/optimal	Sub-optimal/optimal
Clinical reasoning (total)	19.53 (7.11) / 30.11 (4.75)	18.43 (7.20) / 28.77 (5.17)
Clinical decision-making process	10.62 (4.45) / 16.54 (3.04)	10.69 (5.14) / 15.70 (3.35)
Intervention and integration	8.91 (3.53) / 13.57 (2.19)	7.73 (3.19) / 13.07 (2.36)
Midwifery practice (total)	21.53 (5.68) / 31.02 (4.67)	20.02 (5.68) / 30.26 (4.60)
Woman’s relationship with midwife	8.43 (2.36) / 10.64 (1.56)	8.37 (2.59) / 10.58 (1.67)
Midwifery practice	13.09 (4.43) / 20.38 (3.56)	11.65 (4.38) / 19.67 (3.67)

### Internal consistency

Calculated Cronbach’s alpha of the CR total scale, ‘clinical reasoning process’ subscale and ‘intervention and integration’ subscale were 0.93, 0.95 and 0.85 respectively. Calculated Cronbach’s alpha of the MP total scale, ‘women’s relationship with midwife’ subscale and ‘general midwifery practice’ subscale were 0.84, 0.66 and 0.81 respectively.

### Expert panel comments

No members of the expert panels, on returning the packages, asked for specific changes to wording or terminology within the framework. During completion four participants asked for clarification on the meaning of steps in the clinical reasoning process, specifically ‘Cue Acquisition’; ‘Cue Clustering’ and ‘Cue Interpretation’. These were provided in response to those specific requests and the definitions are now provided as part of the post validation Enhancing Decision-making Assessment in Midwifery (EDAM) tool. Comments were more often made on vignettes but consisted of offering advice or stating what the respondent would do in the situation. Six comments were made suggesting that a question was difficult to respond to from the information in the narrative, but there was no consistency across either items or narratives and respondents tended to respond ‘unknown’ to these items.

## Discussion

Findings from this study suggest that the EDAM is a robust, valid and reliable multidimensional psychometric instrument for assessing midwifery decision-making. Further, the invariance testing reveals the instrument to perform in a consistent manner across the different country groups, thus assuring the tool is measuring fundamentally the same construct, in the same way irrespective of cultural context. It appears that the EDAM domains of CR and MP as identified in the qualitative study are valid and robust constructs, which are related but independent of each other. Both domains of EDAM consist of two subscales, which are also distinct but correlated. Both domains and subscales demonstrate excellent psychometric properties including factor structure, model fit and invariance. Whilst these domains (CR and MP) are independent of each other, and hence could be used separately, the known-groups analysis clearly indicates that scores on both scales as well as their embedded subscales discriminate optimal or poor decision-making, supporting the case for combining them under a conceptual model of midwifery decision-making, as proposed in the preceding qualitative work [14, 15, 24]. Qualitative comments from study participants suggest that the tool is able to be utilised effectively in conjunction with ‘real life’ vignettes, however, comments also revealed some limitations in gleaning all the necessary evidence from vignettes. This might suggest that using the EDAM in simulated or reflective situations might have additional value.

**Fig. 2:**
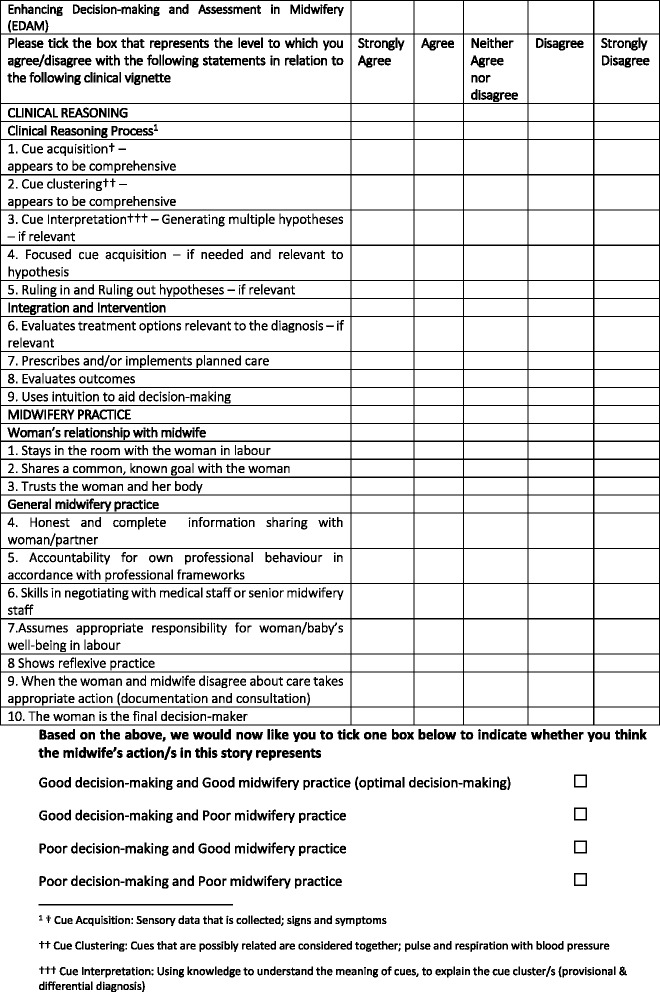
Enhancing Decision-making and Assessment in Midwifery (EDAM) following psychometric evaluation

The original CR and MP scales consisted of 12 and 14 items respectively, following the PCA, five items were initially removed and a further two from the CR scale following the CFA. This reduced the scale overall from 26 to 19 items; 9 in CR and 10 in MP respectively (Fig. 2).

The CR scale mirrors the theoretical perspectives on clinical reasoning theory [22, 23, 44–47] from which it was partially derived, so it is unsurprising that this has emerged as a stable construct within EDAM. Interestingly, however this has split into two clear subscales. The first, ‘the clinical reasoning process’ which seems to focus more on making the decision after balancing the alternatives, as underpinned by hypothetico-deductive theory [48]; the second ‘intergration and interpretation’ on the addition of intuitive thinking and reflection during initiation of the treatment and subsequent evaluation, as proposed within nursing models of clinical reasoning [10, 45] and midwifery applications [49]. Hence both the analysis and existing theoretical base provide strong support for a two factor clinical reasoning scale.

The MP scale also consists of two clear subscales. The first subscale ‘women’s relationship with the midwife’ focuses on the actions necessary to form and maintain the midwife-woman relationship, the second’ general midwifery practice’, is concerned with the behaviours that then occur within that relationship, which support or detract from optimal decision-making. The existing decision-making evidence does not address the effect of context or a person’s wishes for their own health [14]. Professional midwifery and legislative frameworks do however, promote the decisional autonomy of women and a partnership model of working, which is heavily dependent on how the midwife-woman relationship, is both initiated and maintained. Yet this must exist within a context of safety and accountability [2–4]. As decision-making incorporating these elements is a competency outlined in regulatory frameworks, a robust construct which can now be measured by the MP scale within EDAM would seem invaluable in both a practice and an educational context.

Both scales and their related subscales, as already highlighted above, demonstrated good known-groups validity. This evidences EDAMs’ ability to effectively differentiate between optimal and sub-optimal decision-making based on high and low scores and this was a consistent finding across both scales and all four subscales. Whilst no cut-off score defined optimal decision-making in this study, in the context of a validation against a ‘gold standard’ threshold, higher scores were clearly associated with perceived optimal decision-making. Future work where the instrument is tested in the clinical context, would enable determination of threshold scores based on clinical outcomes. Observable difference in the known groups analysis, was the significant differences observed between Australia and the UK with regard to the ‘intervention and integration subscale’ and the ‘general midwifery practice subscale’. Comparison of models by invariance testing revealed no evidence of variance, this scale also revealing excellent measurement and structural invariance characteristics. Confirmation of the invariance characteristics of the EDAM measure emphasises confidence in the statistically significant differences observed between countries not being artefactual of any measurement deficits inherent within the tool but true differences between groups. One possible explanation for this could be that midwifery training and practice engenders all midwives with the base knowledge and learnt skills to follow a clinical reasoning approach to decision-making, albeit in a slightly opaque way, which is largely unaffected by cultural context. However the ability to then apply that knowledge may be more constrained by the cultural context within which midwives practice. The same explanation could be afforded to the country differences identified in the ‘general midwifery practice’ subscale. Whilst midwives across both countries are well equipped to initiate and maintain effective relationships with women, how they are able to facilitate women’s decisional autonomy and negotiate that with other professionals who may become involved in the care may be compromised by the cultural and environment within which they work. The validity of this interpretation will be furnished by wider international work and testing of the instrument within differing cultural contexts of maternity care.

The current study has some limitations. EDAM was generally shown to demonstrate good internal consistency, with one slight caveat. The Cronbachs alpha of the ‘women’s relationship with midwife’ subscale was 0.66, which fails to meet Kline’s [26] criteria of 0.7, the threshold defined for determining good instrument reliability. However, that this subscale consisted of three items, an issue known to deflate Cronbachs alpha values, combined with the fact this alpha is so close to 0.7 potentially makes any concerns about the reliability of this subscale relatively minor. It could be useful in future research to examine the utility of this subscale further.

The EM method of managing missing data, though established as an appropriate approach, nevertheless represents a substitution method based on the profile of non-missing data, thus missing data replaced by this approach is an approximation of the value of the missing data point, thus common to all studies with missing data. The percentage of missing data we managed was relatively small and that we approached the management of it in a considered, systematic, reproducible and established manner, however, this limitation should be acknowledged.

The use of responses from 42 participants, though generating a significant amount of data overall is not an ideal approach. It is possible that despite the vignettes describing different scenarios, that respondents might respond to each vignette set in a characteristically similar manner and that this may impact on the correlation/covariance matrix of the PCA and CFA. It is therefore reasonable to consider the impact of our approach on the measurement characteristics of the EDAM measure in terms of replicability and stability, while acknowledging the content of the data derived from 42 experts in the field. We acknowledge this important limitation of the current study which, though we feel was justified, is nevertheless not typical in EFA and CFA studies and thus represents a compromise between the study design parameters and established statistical convention regarding independence of observations.

A further limitation was the ordering of the vignettes in the pack which was consistent throughout. On reflection, a more methodologically robust approach would have been to use either counter-balancing or random order presentation of the vignettes in the pack to eliminate order effects. It is possible that the consistency of vignette ordering in the current study could have influenced participant responding in a systematic way and be a potential source of confound. While acknowledging this possibility, we are minded of the distinctiveness of each vignette and would anticipate that potential order effects to be minor. However, future studies would benefit considerably from remedy of our methodological oversight by utilising a random order or counterbalanced presentation of the vignettes. Future research is therefore recommended and encouraged, in larger samples, to further confirm a stable factor structure, reliability and validity of the tool. Future research would also be required to collect data suitable to enable divergent validity to be evaluated as an additional and valuable index of validity.

One further issue of note, is the use of the term ‘if relevant’ in 4 of the EDAM questions, which could be considered ambiguous to respondents or lead to a forced answer. This was not raised as an issue by the panels, these questions were consistently responded to and missing data for these questions was both minimal and randomly distributed across the data set. However, it should be acknowledged as an area that future work on EDAM needs to consider more fully how to deal with.

Despite the limitations of this study, that the instrument performs well in a cross cultural context offers exciting opportunities for its use as both a framework and assessment tool, for the delivery and efficacy of education and training around decision-making in both students and qualified midwives. It also has the potential to determine the relationship of optimal decision-making to improved clinical outcomes and safe, high quality care. Applying the tool in the context of an actual event for a midwife might be challenging, but embedding the steps and principles through education, training and reflective practice, could effectively support decision-making in practice. Standardised guidance for midwives’ in the labour and birth period has the potential to reduce inconsistencies in care delivery, improve the quality of midwives’ relationships with women and other care providers, and enhance midwives’ ability to practice effectively and with confidence.

Ultimately it is hoped that EDAM could facilitate evaluation in relation to clinical outcomes. This could have global utility and significance in clinical and research terms. Further research to test the instrument in other cultural contexts beyond Australia and the UK, would be of significant value.

## Conclusions

The EDAM appears to provide a sound psychometric instrument for assessing midwifery decision-making. It offers the opportunity to robustly assess midwifery decision-making combining both a cognitive clinical reasoning component that offers room for intuitive thinking, reflection and incorporation of midwifery philosophy, alongside the necessity of an effective relationship with the woman, as a partner in her care. This offers the advantage of standardising decision-making processes and practices, demonstrating transparent and defensible decision-making, alongside adherence to the regulatory and legislative demands of the profession. This promotes a model of shared decision-making that facilitates the autonomy of women during labour and birth.

## Additional file

Additional file 1: Midwifery experience characteristic of expert panels (PDF 324 kb) Additional file 2: Vignette example (PDF 266 kb)

## References

[1] International Confederation of Midwives. Global Standards for Midwifery Regulation. International Confederation of Midwives; 2010 (amended 2013). http://internationalmidwives.org/assets/uploads/documents/CoreDocuments/ICM%20Standards%20Guidelines_ammended2013.pdf. Accessed 7 Apr 2015.

[2] Nursing and Midwifery Board of Australia (2006). National Competency Standards for the Midwife.

[3] Nursing and Midwifery Board of Australia (2008). Code of Professional Conduct for Midwives in Australia.

[4] Nursing and Midwifery Council (2009). Pre-registration Standards for Midwifery education.

[5] Nieuwenhuijze MJ, Korstjens I, de Jonge A, de Vries R, Lagro-Janssen A. On speaking terms: a Delphi study on shared decision-making in maternity care. BMC Pregnancy Childbirth. 2014; http://www.biomedcentral.com/content/pdf/1471-2393-14-223.pdf. Accessed 10 Dec 2014 10.1186/1471-2393-14-223 PMC4104734 25008286

[6] Kitzinger S (2005). The Politics of Birth.

[7] Elwyn G, Frosch D, Thomson R, Joeseph-Williams N, Lloyd A, Kinnersley P (2012). Shared decision-making: a model for clinical practice. J Gen Intern Med.

[8] Terekina A, Borcherding K, Larichev OI, Messick DM (1990). Knowledge organization studies through analysis of cognitive patterns. Contemporary issues in decision-making.

[9] Croskerry PA (2009). Universal Model of Diagnostic Reasoning. Acad Med.

[10] Levett-Jones T, Hoffman K, Dempsey J, Jelong S, Noble D, Norton C, Roche J, Hickey N (2009). The ‘five rights’ of clinical reasoning: An educational model to enhance nursing students’ ability to identify and manage clinically ‘at risk’ patients. Nurs Educ Today.

[11] Standing M, Standing M (2010). Cognitive Continuum Theory-Nine modes of practice. Clinical Judgement and Decision-Making in Nursing and Interprofessional Healthcare.

[12] Mok H, Stevens P, Raynor M, Marshall J, Sullivan A (2005). Models of Decision-making. Decision-making in Midwifery Practice.

[13] Jefford E, Fahy K, Sundin D (2011). Decision-Making Theories and their usefulness to the midwifery profession both in terms of midwifery practice and the education of midwives. Int J Nurs Pract.

[14] Jefford E (2012). Optimal Midwifery Decision-Making during 2nd Stage labour: The integration of Clinical Reasoning into Practice.

[15] Jefford E (2014). The Midwife and Decision-Making Processes: Integration into clinical practice.

[16] Royal College of Obstetricians and Gynaecologists (2011). High Quality Women’s Health Care: A proposal for change.

[17] Hutton J. COR 2008/647. John Hutton. Coroner’s; 2011

[18] Nursing and Midwifery Board of Australia (2010). Midwifery Practice Decision Flowchart.

[19] Page L, Hutton E, Page L, Percival P (2000). Introduction: Setting the scene. The new midwifery: Science and sensitivity in practice.

[20] Elstein AS, Shulman LS, Sprafka SA (1978). Medical problem solving: An analysis of clinical reasoning.

[21] Elstein AS, Bordage GC, Dowie J, Elstein AS (1988). Psychology of Clinical Reasoning. Professional judgment: A reader in clinical decision-making.

[22] Thompson C (1999). A Conceptual Treadmill: The need for ‘middle ground’ in clinical decision-making theory in nursing. J Adv Nurs.

[23] Thompson C (1999). Pearls, pith, and provocation: Qualitative research into nurse decision-making: factors for consideration in theoretical sampling. Qual Health Res.

[24] Jefford E, Fahy K (2015). Midwives’ clinical reasoning during 2nd stage labour: Report on an interpretive study. Midwifery.

[25] Cresswell J, Plano Clark V (2011). Designing and Conducting Mixed Methods Research.

[26] Kline PA (2000). Psychometrics Primer.

[27] Kline P (1993). Handbook of Psychological Testing.

[28] Spiteri MC, Jomeen J, Martin CR (2013). Reimagining the General Health Questionnaire as a measure of emotional wellbeing: a study of postpartum women in Malta. Women Birth.

[29] Byrne BM (2010). Structural equation modeling with AMOS: Basic concepts, applications, and programming.

[30] Hollins Martin CJ, Martin CR (2014). Development and psychometric properties of the Birth Satisfaction Scale-Revised (BSS-R). Midwifery.

[31] Bentler PM, Bonett DG (1980). Significance tests and goodness of fit in the analysis of covariance structures. Psychol Bull.

[32] Bentler PM (1990). Comparative fit indexes in structural models. Psychol Bull.

[33] Hu LT, Bentler PM, Hoyle RH (1995). Evaluating model fit. Structural Equation Modeling: Concepts, Issues and Applications.

[34] Hu LT, Bentler PM (1999). Cutoff criteria for fit indexes in covariance structure analysis: conventional criteria versus new alternatives. Struct Equ Modeling.

[35] Browne MW, Cudeck R, Bollen KA, Long JS (1993). Alternative ways of assessing model fit. Testing Structural Equation Models.

[36] Schumacker RE, Lomax RG (2010). A beginner’s guide to structural equation modelling.

[37] Hooper D, Coughlan J, Mullen MR (2008). Structural equation modelling: Guidelines for determining model fit. EJBRM.

[38] Asparouhov T, Muthén B (2009). Exploratory structural equation modelling. Struct Equ Model.

[39] SPSS (2009). PASW Statistics 18 Core System User’s Guide.

[40] SPSS (2009). PASW Advanced Statistics 18.

[41] Arbuckle JL. AMOS 18 User’s Guide. Chicago, IL: AMOS Development Corporation; 1995–2009.

[42] Redshaw M, Martin C, Rowe R, Hockley C (2009). The Oxford Worries about Labour Scale: Women’s experience and measurement characteristics of a measure of maternal concern about labour and birth. Psychol Health Med.

[43] West R (1991). Computing for Psychologists.

[44] Higgs J, Jones M, Loftus S, Christensen N. Clinical reasoning in the health professions. Amsterdam: Butterworth Heinemann; 2008

[45] Benner P (1984). From Novice to Expert: Excellence and Power in Clinical Nursing Practice.

[46] Mong-Chue C (2000). The challenges of midwifery practice for critical thinking. Br J Midwifery.

[47] Offredy M (2002). Decision-making in primary care: Outcomes from a study using patient scenarios. J Adv Nurs.

[48] Newell A, Simon HA (1972). Human problem solving.

[49] Raynor M, Marshall J, Sullivan A (2005). Decision-making in Midwifery Practice.

