# Supplementary material for: Determining the psychometric properties of the Enhancing Decision-making Assessment in Midwifery (EDAM) measure in a cross cultural context
Source: BMC Pregnancy Childbirth. 2016 Apr 28;16:95. doi: 10.1186/s12884-016-0882-3 (PMC4850679; doi:10.1186/s12884-016-0882-3)
Supplement: Additional file 1: — Midwifery experience characteristic of expert panels (PDF 324 kb) [file 12884_2016_882_MOESM1_ESM.pdf]

Australian Participants	Maternity Service	Years of Experience	UK Participants	Maternity Service	Years of Experience
1	Public Hospital Tertiary Metropolitan	33	1	District General Hospital/SOM*	20
2	Public Hospital Tertiary Regional	24	2	District General Hospital/SOM*	26
3	Public Hospital Secondary Metropolitan	17	3	City Consultant Led Care	6
4	Public Hospital Secondary Regional	9	4	City Consultant Led Care	20
5	Public Hospital Secondary Rural	29	5	Head of Midwifery/ City Consultant Led Care acute and alongside Midwifery Led Unit/SOM*	30
6	Public Hospital Secondary Remote	13	6	City alongside Midwifery Led Unit	18
7	Birth Centre attached to public hospital	40	7	City Consultant Led Care acute and alongside Midwifery Led Unit/SOM*	25
8	Birth Centre free standing	29	8	Rural Hospital	18
9	Group Midwifery Practice attached to public hospital	2	9	Community Midwife	10
10	Caseload Midwifery Practice attached to public hospital	9	10	Rural Community Midwife	21
11	Private Maternity Unit Metropolitan	15	11	Rural Community Midwife	20
12	Private Maternity Unit Regional	28	12	Remote Community Midwife	15
13	Private Maternity Unit Rural	38	13	Caseload Holding Midwife	12
14	Independent Midwife (Private Practice)	33	14	Consultant Midwife	17
15	Eligible Midwife	11	15	Consultant Midwife	40
16	Midwifery Practice attached to a private hospital	U/K	16	Academic	32
17	NGO Aboriginal Birth Services Metropolitan	5	17	Academic/Professor of Midwifery	22
18	NGO Aboriginal Birth Services Regional	32	18	Independent Midwife (withdrew)	
19	NGO Aboriginal Birth Services Rural	36	19	Consumer (withdrew)	
20	NGO Aboriginal Birth Services Remote	35			
21	Academic	22			
22	Academic	34			
23	Academic/ Private Maternity Unit Metropolitan	30			
24	Consumer	N/A			
25	Consumer	N/A			
