# Supplementary material for: Determining the psychometric properties of the Enhancing Decision-making Assessment in Midwifery (EDAM) measure in a cross cultural context
Source: BMC Pregnancy Childbirth. 2016 Apr 28;16:95. doi: 10.1186/s12884-016-0882-3 (PMC4850679; doi:10.1186/s12884-016-0882-3)
Supplement: Additional file 2: — Vignette example (PDF 266 kb) [file 12884_2016_882_MOESM2_ESM.pdf]

**ADELAIDE**
**Background to Adelaide's Story**
Adelaide has worked for 33 years as a midwife. She is currently working in a birth centre attached to a public hospital located in a major city. When the birth unit is quiet, Adelaide is required to work on the hospital's delivery unit where this story occurred.
Adelaide arrived at 7 am and began caring for Rosalie, a primigravida. Rosalie had laboured all through the night and had been diagnosed, two hours previously, as being in 2nd stage labour. Rosalie had an epidural, a CTG and had been actively pushing following verbal cues from the midwives since full dilation. The whole family were aware that the doctors were planning to conduct an assisted birth shortly.
**Adelaide's Story**
When I entered Rosalie's birthing room, I spent a few minutes getting to know Rosalie, Mike and Joan. I tried to engage them in conversation and basically get a feel of how Rosalie was feeling about the way her labour and birth was progressing. Whilst this was happening, I used all my senses and assessed everything. I looked at Rosalie's position on the bed, her colour, whether she was perspiring, whether she was exhausted or was she happy or sad? In essence, I assessed her general well being and her mental state. I looked at the support people and asked myself, are they in a chair snoring? Are they exhausted from supporting Rosalie all night?' I also assessed the room for clutter or whether it was disorganised. I saw, when Rosalie was pushing, there was a **tiniest little bit** (*emphasis*) of fetal head on view, but Rosalie was so exhausted after labouring all night that her pushing was not effective at progressing delivery of her baby. I knew the doctors, [once the shift changed in approximately 30 minutes], would come and do an assisted birth.
As Rosalie was pushing with each contraction, I sensed Rosalie did not want to throw the towel in and have an assisted birth. I thought, in this instance, I had to do something; **we** (*emphasis*) somehow had to push this baby out unassisted. I knew that if I got the lithotomy poles adjusted to suit Rosalie's anatomy and she could just let her knees flop whilst supporting behind the back of her legs she would be able to get much more 'push' into her bottom and get this baby round the bend of the pelvis. My decision was based on looking at the whole picture of Rosalie and my knowledge of the body. I've observed placing the woman in the lithotomy position worked quite a few times in my experience and from observing other midwives**.** Once I'd made my decision, I actually said to Rosalie, "would you mind very much if I put your legs into a lithotomy? I explained to her what I wanted to do.
**Outcome**
Within twenty minutes of Rosalie agreeing to use the lithotomy poles she birthed her child unassisted.
